# Models Applied to Grapevine Pests: A Review

**DOI:** 10.3390/insects12020169

**Published:** 2021-02-16

**Authors:** Federico Lessio, Alberto Alma

**Affiliations:** DISAFA, Entomology Unit, University of Torino, Largo Braccini 2, 10095 Grugliasco (TO), Italy; federico.lessio@unito.it

**Keywords:** grapevine, pest management, modelling, decision-support system, *Xylella fastidiosa*, *Lobesia botrana*, *Scaphoideus titanus*

## Abstract

**Simple Summary:**

Mathematical models are developed to predict key aspects of insects harmful to many crops, including grapevine. Practical applications of these models include forecasting seasonal occurrence and spread over space in order to make decisions about pest management (e.g., timing of insecticide sprays). Many models have recently been developed to evaluate the spread of insect pests on grapevine under a climate change scenario as well as to forecast the possibility that alien species could settle into new environments. To make the published models available to vine-growers and their stakeholders, a holistic approach presenting these models within the frame of a decision support system should be followed.

**Abstract:**

This paper reviews the existing predictive models concerning insects and mites harmful to grapevine. A brief conceptual description is given on the definition of a model and about different types of models: deterministic vs. stochastics, continuous vs. discrete, analytical vs. computer-based, and descriptive vs. data-driven. The main biological aspects of grapevine pests covered by different types of models are phenology, population growth and dynamics, species distribution, and invasion risk. A particular emphasis is put on forecasting epidemics of plant disease agents transmitted by insects with sucking-piercing mouthparts. The most investigated species or groups are the glassy-winged sharpshooter *Homalodisca vitripennis* (Germar) and other vectors of *Xylella fastidiosa* subsp. *fastidiosa*, a bacterium agent of Pierce’s disease; the European grape berry moth, *Lobesia botrana* (Denis and Schiffermuller); and the leafhopper *Scaphoideus titanus* Ball, the main vector of phytoplasmas agents of Flavescence dorée. Finally, the present and future of decision-support systems (DSS) in viticulture is discussed.

## 1. Introduction

Arthropod pests cause yearly heavy losses in viticulture. The cost of pest management is constantly increasing due to a lack of active ingredients, the introduction of alien species, and so on. Optimization of pest management strategies is therefore a key point in viticulture. Pest management in viticulture should follow an integrated approach, including chemical sprays, biological control agents, agroecology, mating disruption, and forecast models [1]. The latter represent a useful set of tools, as they allow us to make timely decisions in targeting pests or considering a particular factor of risk. The main (ultimate) purpose of a model in agriculture is to produce a pest-management decision-support system (DSS) [2,3]. This fits phenological and demographical models, which allow for a forecast of insect population dynamics over time, permitting to drive insecticidal sprays [4]. Another frequently covered issue is risk assessment, for which the output of the model is often a risk map [5,6]. The geographical displacement of insect pests is also a stressed point within the frame of a climate-changing scenario. Nevertheless, bringing the potential benefit of using modelling tools to the stakeholders (e.g., extension services and farmers) often represents a challenge to scientists [3]. Although many models in grapevine entomology are about single species, the future of modelling in pest management should be directed towards comprehensive frameworks, embracing more aspects of the problem, e.g., economics, crop yields, etc., in which the insect pest becomes a part of the whole [6]. For instance, it may become necessary to make a choice about timing of insecticidal sprays against two different pests overlapping within the season, and if both pests are forecasted by a model, it would be easier to choose the best active ingredient, making the decision eco-friendlier and cheaper.

In this review, we go over the state-of-art of the different models designed and applied to insect pests of grapevines worldwide, investigating their utilities and limits and defining their actual and potential applications in decision-support systems (DSS). While the main part of the species overviewed are modelled with exact reference to grapevine, some of them have been modelled referring to other host plants but are listed here since such pests can be harmful to grapevine too. We start with a brief review of the basic concepts of modelling and then list the different types of models that have been implemented with regard to grapevine insect pests. Finally, we focus on the building and application of different types of models for some of the most important grapevine pests or diseases transmitted by pests. The literature was reviewed using the search engines Scopus, Web of Science, and Google Scholar, and the search period ranged from 1990 to 2020. The following keywords (listed here alphabetically) were used in multiple combinations: berry moth(s), calibration, climate change, decision-support system, demographic, deterministic, entomology, epidemiology, *Eupoecilia ambiguella*, insects, invasion risk, leafhoppers, *Lobesia botrana*, mealybugs, model (modelling), grape, grapevine, parametrization, pest, pest management, phenology, phytoplasmas, Pierce’s disease, population dynamics, prediction, *Scaphoideus titanus*, spatial distribution, spider mites, stochastic, validation, vectors, viticulture, *Vitis vinifera,* and *Xylella fastidiosa*. Usually, a combination included one keyword related to grapevine (e.g., *Vitis*, grapevine, etc.), one keyword related to the pest (e.g., spider mites), and one keyword related to modelling (e.g., modelling, prediction, etc.). When the searched pest was a grapevine specialist (e.g., *Scaphoideus titanus*), the keyword related to grapevine was omitted.

## 2. What Is a Model?

The definition of a mathematical model is a description of a system using mathematical concepts and language to facilitate proper explanation of a system or to study the effects of different components and to make predictions [7]. The term “modelling” refers to the building process of a model itself and includes the following steps: identify what is important, list the quantities that can be observed (outputs), list the variables that can be controlled (inputs), and define the constraints of the system [8]. 

Mathematical models may be divided into deterministic and stochastic. Deterministic models are defined by the parameters and the initial conditions chosen only; many of them are based on ordinary differential equations (ODEs). On the other hand, stochastic models include a certain rate of randomness; models derived from data fitting are typically stochastic [9]. Models may also be divided into continuous and discrete depending on the type of output variable [9]. In addition, we can discern between analytical and simulation (computer) models [9].

Finally, descriptive or process models are as such when they explicitly incorporate biological aspects of the phenomenon investigated. The opposite is statistical models, derived solely from data fitting: whilst many of them are useful in identifying patterns and other biological phenomena, statistical models are not conceptually based and therefore give no *a priori* explanation [9]. Nevertheless, statistical models are used often to validate the results of process modelling, fitting them to independent data. Given the wide array of statistical models concerning insect pests in viticulture, they are not covered by this review.

## 3. Predicted Phenomena in Entomology

### 3.1. Pest Population Growth and Dynamic

Developmental (phenology) models are usually based on temperature, which is the main abiotic factor driving the physiological response of poikilothermic organisms, including insects [6,10]. The first such models were built using degree days (DD) and minimum cardinal temperatures [11,12]. These models were mostly linear; however, the response to temperature by insects has a high nonlinear component. A temperature-dependent nonlinear growth function was theorized by Logan et al. [13] and subsequently improved by Briére [10]. This function models the developmental rate of arthropods under constant temperatures by estimating minimum, optimum, and maximum temperatures of development. Concerning grapevine pests, it has been applied to a leafhopper [14] and to the grape berry moth [15]. This and other models applied to these pests are discussed later. 

A further improvement on this issue is given by distributed delay models (DDMs). These models are derived from both phenological and demographical models and are used when the response to a driven factor is not immediate but happens with a given delay [16,17,18]. This is typical of insect developmental dynamics, as different individuals do not behave as a cohort but they grow and molt at different times, overlapping themselves. DDMs are therefore typically continuous models [6]. Thinking in population rather than single individual terms, we have from age-structured to stage-structured models [18,19,20]. Stage-structured models are particularly helpful in entomology, as population dynamics of insects involve different life stages with different growth rates, responses to external stimuli, and so on. In pest-management terms, predicting the occurrence of a particular life stage is crucial for timing sprays, release of a natural enemy, and so on. In viticulture, this is particularly interesting concerning *Scaphoideus titanus* Ball (Hemiptera: Cicadellidae) for targeting third instar nymphs to avoid phytoplasmas’ acquisition [21,22].

### 3.2. Pest Invasion Risk

Another issue needing investigation is not “when” but “where” an insect pest occurs. In this case, what matters is not time, but space. The occurrence of an insect pest in space could be investigated at different scale levels: plot-scale (e.g., a single vineyard), landscape scale (a vine-growing area), country-scale, and so on. The importance of landscape ecology in pest management has increased over the last 15 years, as the problem has been more frequently approached at the landscape- rather than plot-scale. Spatial distribution models are relatively recent, but connected research is increasing exponentially since the birth of Geographic information system (GIS)-based tools, which allow us to handle georeferenced data [23].

Many of these models aim to forecast the global distribution of pests under a climate change scenario. Pest-risk assessment is crucial in forecasting the introduction and, more importantly, the establishment of alien species into a new area in order to put in place plant protection procedures. The influence of climate change on viticulture involves the host plant, insect pests, and their natural enemies [24]. The so-called species’ distribution models (SDMs) include ecological niches (e.g., bio-climatic models, BIOCLIM), a generic algorithm for rule set prediction, maximum entropy, and CLIMEX (Climate Modelling of Extreme Events) models. Ecological niche models (ENMs) represent an approximation of the species’ realized niche (resulting in its occupied geographical space) and not of its fundamental niche (ecological space). However, if the fundamental niche is adequately represented, the projection of the model into geographical space represents the potential species’ distribution [25]. An evolution of ENM is given by physiologically based demographical models (PBDMs) [26]. CLIMEX, GARP (genetic algorithm for rule-set prediction) models are machine-learned, stochastic processes using presence data only. They search broadly within the search space and then refine solutions showing high values of optimization criteria [27]. Maximum entropy (MAXENT) models are intended to make predictions from incomplete information. They estimate a target probability distribution by finding the probability distribution of maximum entropy given a set of constraints representing the incomplete information about our target distribution. MAXENT are machine-learning models working with presence data only [25,28]. CLIMEX models, on the other hand, are entirely based on climatic factors. The inference is that simple: given the current distribution of a species, the required climatic conditions can be inferred [29,30].

## 4. Modelling Grapevine Pests

**Figure 1:**
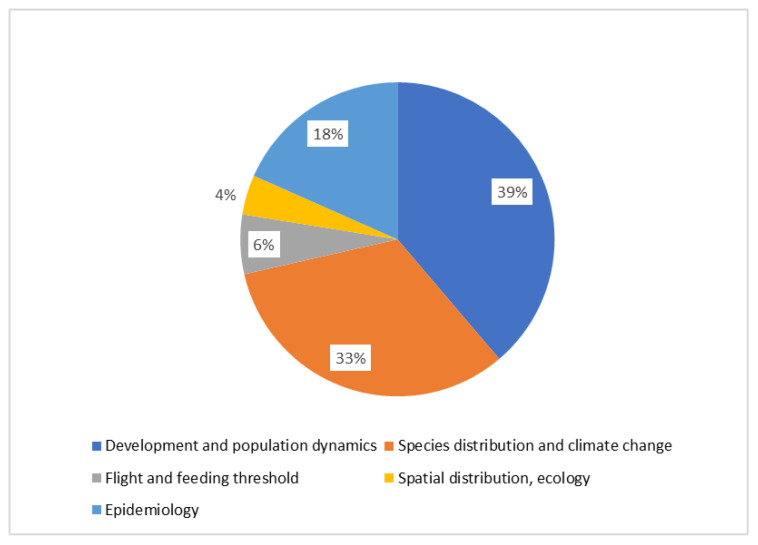
Distribution of published models investigating different life aspects of grapevine pests.

**Figure 2:**
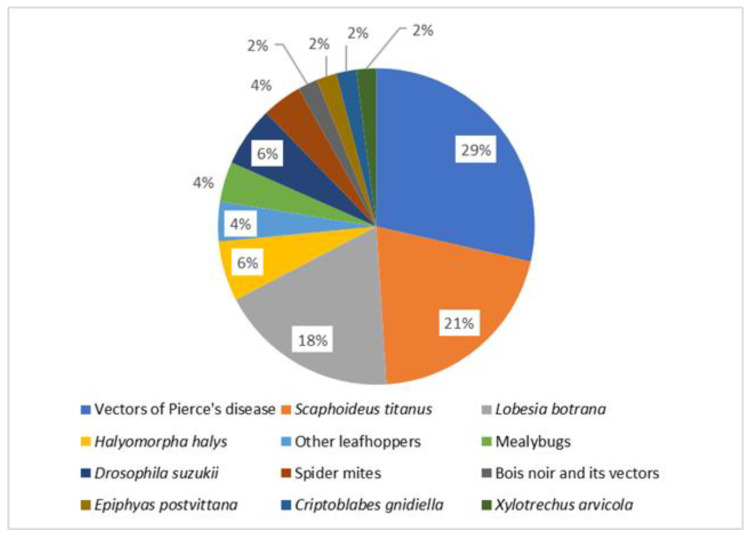
Distribution of published models investigating different taxa of grapevine pests.

A list of the available models investigating different aspects of grapevine pests is presented in Table 1. The most covered aspect is development and population dynamics (38%), followed by species distribution and climate change (33%) (Figure 1). Concerning different taxa, the vectors of *Xylella fastidiosa* are the most represented (29%), followed by the European grape berry moth (21%), and *S. titanus* (19%) (Figure 2). These three groups are presented in detail as case studies, whereas the others are covered after.

### 4.1. Case Study 1: Grapevine Yellows and Their Vectors

Diseases caused by phytoplasmas, commonly referred to as “yellows”, transmitted by insect vectors are among the most threatening to grapevine. Since so far there is no cure, particular attention has been paid to vector control. The most important are Flavescence dorée (FD) and Bois noir (BN), which are transmitted by different vector species [21]. From a modelling point of view, we can distinguish between models referring to aspects of vector biology only and models that encompasses both disease and vector. The majority of models are about FD and/or its main vector *S. titanus*. 

The first models concerning *S. titanus* aimed to forecast the appearance of nymphs and adults. Rigamonti et al. [22] developed a stochastic model to forecast the appearance of N1, N3, and adults for insecticide timing (especially insect growth regulators). This model is currently used in Switzerland within adaptative management (AM) strategies. Subsequently, another stochastic model for the multiannual infestation pattern of *S. titanus* on grapevine was derived from this one [72]. On the other hand, the duration of all life stages in *S. titanus* (including eggs) at constant temperatures was studied basing on Briére’s equations, and the obtained deterministic model was applied to field conditions using R software [14]. A more comprehensive stochastic, stage-structured model for *S. titanus* populations was developed taking into account input variables other than temperature, such as plant density and others [20].

Spatial models on *S. titanus*, on the other hand, have been less investigated. These aspects have been studied mainly by means of spatial interpolation techniques such as geostatistical analyses [73,74]. However, a study was conducted to forecast the spread of *S. titanus* at the local scale using an Artificial Neural Network (ANN) [54]. ANNs are machine self-learning processes, inspired to brain connections, which estimate one or more variables from a given data set [6]. 

Other models related to *S. titanus* concern species’ distribution and climate change. A survey was made with DIVA-GIS software to forecast the possible spread of *S. titanus* in China [50], and another one was made in Chile using BIOCLIM [52]. Within the frame of a climate change scenario, the adaptation of *S. titanus* to an alpine environment was also investigated [51]. Finally, VITISCLIM (“*Vitis* and Climate”) model was applied in Austria [53].

Epidemiological models were also developed in an attempt to forecast the spread of FD. A stochastic model, based on [20], was developed to model the acquisition, latency, and inoculation process of FD phytoplasmas in grapevine [75]. On the other hand, a deterministic model was developed to forecast the FD epidemic without referring directly to the vector’s population, which is modelled by means of a coupling factor between healthy and infected plants; moreover, this model takes into account the presence of hotbeds, levels of susceptibility of grapevine varieties, and insecticidal sprays [55]. Concerning Bois noir (BN), a model about the long-term response of grapevines to the pathogen was developed using data mining and decision trees [49]. However, this is not an epidemiological but a prognosis model in that it does not model the pattern of disease transmission. It must be said, however, that BN phytoplasma “Ca. *Phytoplasma solani*” has many insect vectors, depends on many environmental variables, and is therefore very difficult to model from an epidemiological point of view [76]. 

Finally, an approach attempted to model the spread of FD from economic and social points of view [77]. Additionally, a model to evaluate the profitability of replacing symptomatic plants was developed [78]. Modelling the economic impact of pest and/or plant disease management is always a challenge and has been overlooked so far [6].

### 4.2. Case Study 2: Grape Berry Moths

The European grape berry moth *Lobesia botrana* (Denis and Schiffermuller) is probably one of the most studied pests of grapevine concerning the development and applications of mathematical models. The first models and their evolutions were based on degree days, aimed at predicting flight [11,12,15,63,79]. A deterministic model was also developed to calculate diapause length, including the duration of pre- and post-diapausing larvae and diapausing pupae depending on temperature and photoperiod [80]. A stage-structured population model based on partial differential equations (PDAs), permitting to distinguish growth of individuals within a cohort, was also developed: the environmental factors included air temperature, relative humidity, and grape variety [81]. A holistic, physiologically based, demographic model (PBDM), based on a grapevine model with subunits for the growth of leaves, shoots, clusters, and so on and a *L. botrana* age-structured model, was also developed. This model includes bottom-up and top-down effects [62]. Based on the model by Gutierrez et al. [62], a PBDM was developed to forecast the geographical distribution of the grape berry moth, both present and under a climate change scenario [59]. Another PBDM was based on a stochastic demographic model for a stage-structured population and aimed to support integrated pest management (IPM) strategies [4]. In addition, a MAXENT model was developed to forecast the potential distribution of *L. botrana* in China [60].

### 4.3. Case Study 3: Pierce’s Disease and Its Vectors

Pierce’s disease (PD) of grapevine is caused by a bacterium, *Xylella fastidiosa* subsp. *fastidiosa.* There are many strains (subspecies) of *X. fastidiosa* causing different syndromes to many host plants [82]. It is worth remembering that *X. fastidiosa* subsp. *pauca*, recently identified in Apulia, Southern Italy, caused the severe decline syndrome named CoDiRo to olive trees [83]. *X. fastidiosa* is transmitted by xylem-feeding insect vectors such as spittlebugs and sharpshooters [84,85]. Some of them have been investigated from a modelling point of view, with or without particular reference to PD, and are reported here.

A first set of papers modelled the physiology and demography of vectors. The most studied vector was the glassy-winged sharpshooter (GWSS) *Homalodisca vitripennis* (Germar) (*H. coagulata* (Say)), concerning embryonic development [86], development at constant temperatures, and subsequently its adaptation to Californian climate [37], and population dynamics [34]. Additionally, a temperature-dependent stochastic phenology model was developed [38]. In terms of epidemiology, the patterns of transmission of PD by *H. vitripennis* were modelled with a discrete-time simulation model [44]. Another research, albeit not involving vectors, modelled the response to cold by grapevine infected by *X. fastidiosa* [46], whereas the research proposed by Kyrkou et al. [43] modelled the PD epidemiology depending on infected vines and GWSSs (in the authors’ words, the dynamics of vine population under a high PD pressure). A temperature-dependent feeding model of the GWSS was developed to forecast probing [41]. *A. latere* modelling concerned also the development of an egg parasitoid of the GWSS [87]. In terms of species’ distribution, both PD [39] and GWSS [26] were investigated. The potential spread of three different subspecies of *X. fastidiosa*, namely *fastidiosa, pauca,* and *multiplex*, were studied with a species distribution model [39].

Other acknowledged vectors of PD are less investigated, probably because GWSS is the only species overwintering in the adult stage and capable therefore of propagating the disease from year to year [84]. In Europe, the common spittlebugs *Philaenus spumarius* L. and *Neophilaenus campestris* L. have been associated to CodiRo, and *P. spumarius* has been acknowledged as a vector [85]. However, to date, there is no or little literature about modelling life aspects of spittlebugs besides a study about the influence of temperature on *Neophilaenus* sp. [35].

### 4.4. Other Species

One of the main emerging pests is the brown marmorated stink bug (BMSB), *Halyomorpha halys* (Stål) (Hemiptera: Pentatomidae). A temperature-dependent model was implemented to forecast oviposition [31], whereas other models are about its potential distribution [32,33]. Within coleopterans, the potential spread of *Xylotrechus arvicola* (Oliver) (Coleoptera: Cerambycidae) in Spain following climate change scenarios was studied by means of a degree-days-based model [66]. Concerning mealybugs, which are important pests and vectors of viruses [24], a forecast model about the spread of *Planococcus ficus* Signoret in California, including pest phenological and demographical aspects, as well as the physiology of grapevine and the influence of climate on natural enemies was developed [56], whereas a spatial model was proposed to forecast spatial distribution of leaf roll affected grapevines [57]. The spotted-winged *Drosophila suzukii* Matsumura (Diptera: Drosophilidae) is another pest of some concern on grapevine [88]. However, to date, there are no models developed with respect to its relationships with grapevine. A generalized additive model (GAM) was used to predict flight based upon some environmental and weather factors, namely some T and RH parameters: this model was developed with respect to blueberry [67], whereas a SDM modelled its expansion range [69]. Recently, a physiological model, based on the equations of Logan and Briére, was also published: this model forecasted the development, fertility, and mortality of *D. suzukii* [68].

Spider mites (Arachnida: Acari) are another important category of pests of grapevine. However, they are scarcely explored from a modelling point of view, as a data-fitting approach is usually preferred. The most investigated matter is prey vs. predator interaction. Such an approach was applied to *Tetranychus urticae* Koch and a predatory mite using the Penna model [89]. Another stage-specific predation model was developed with respect to *T. urticae* and *Phytoseiulus persimilis* Athias-Henriot [90]. A temperature-dependent model according to Logan [13] was developed with respect to the yellow mite *Eotetranychus hirsti* Pritchard and Baker [71], and a degree-days-based model was used to predict the oviposition by *T. urticae* [70]. Finally, an empirical transition matrix model between *Panonychus ulmi* Koch and two predatory mites belonging to the families Phytoseiidae and Stigmeidae was proposed [91].

## 5. Decision Support Systems: Present and Future

Decision support systems (DSS) should be the final purpose of a model designed for pest management in viticulture; that is where such a model should end, being available to vine growers, wine makers, technical support professionals, plant-protection services, and other stakeholders. Underutilization in practical agriculture is one of the main issues in mathematical models [2]. Another problem is the compartmental approach that drives many applications of models [6]. Some models are currently used, e.g., the “Modello *Lobesia*” in Piedmont, Italy, to forecast the European berry moth [92]. However, a holistic approach, including meteorology, insect phenology, demography and spatial distribution, plant phenology, and the economic impact of choices made by vine growers, is desired. For instance, in order to make existing models available to stakeholders, a portal (a website) encompassing different compartments adapted to different vine growing areas, organic vs. conventional pest management, plant phenology vs. bud break, and blossoming/flowering depending also on vine variety, may be developed. Moreover, a distinction between short- and long-term decision-support systems should be taken into account. Short-term decisions are made within season (e.g., timing of insecticidal sprays), whereas long-term decisions have to do with the choice of area when making new plantations. This latter issue is particularly critical when dealing with plant diseases transmitted by vectors: the prediction of a long-term effect of surrounding hotbeds on a new vineyard may drive the choice of a given geographical area [55]. Of course, models should be included into the frame of integrated pest management along with other methods or techniques such as sampling plans [93,94]. 

The website may be structured in a way to allow scientists to directly upload recently published research, and a search function within the website would then allow users to retrieve information promptly and to make decisions, for instance, about choosing the optimal timing to target two or more pests having partially overlapping life cycles with one single spray of insecticide given the temperature-dependent population dynamics simulated by the models or putting together spread potential models, thermal threshold models, and population growth models to forecast the acclimation and expansion of an alien species over a non-infested areas in order to make decisions about the extension of buffer zones by plant-protection services. 

The greatest challenges are probably the climate change scenarios (particularly global warming) and the potential introduction of alien species into different viticultural areas. However, these two issues differ in terms of increase over time. Global warming occurs gradually but is almost certain and is therefore easy to simulate and forecast over the long period: from an entomological point of view, some grapevine pests may spread farther northwards or above sea level [51,59] along with grapevine itself [95,96]. On the other hand, the introduction of alien species is often an abrupt event, requiring quick adaptations: from this point of view, it is very important to model the potential adaptation of alien species to new viticultural areas well before they are detected [50,52] in order to consider in advance the required countermeasures.

In conclusion, the literature on prediction models about pests of grapevine is wide and adequately covers the most important biological and epidemiological aspects, but an effort to put together this knowledge and to make it useful to vine growers and their stakeholders is needed.

## Figures and Tables

**Table 1:** List of published models investigating different biological aspects of grapevine pests.

Order	Species	Development and Population Dynamics	Species Distribution and Climate Change	Flight and Feeding Threshold	Spatial Distribution, Ecology	Epidemiology
Hemiptera	*Halyomorpha halys*	[31]	[32,33]			
	Vectors of *Xylella fastidiosa*	[34,35,36,37,38]	[26,39,40]	[41]	[42]	[43,44,45,46]
	Other leafhoppers/planthoppers	[47,48]				
	Bois noir and its vectors					[49]
	*Scaphoideus titanus*	[14,22]	[50,51,52,53]		[54]	[20,53,55]
	Mealybugs		[56]			[57]
Lepidoptera	*Lobesia botrana*	[11,15,58]	[59,60,61,62]	[12,63]		
	*Epiphyas postvittana*		[64]			
	*Criptoblabes gnidiella*	[65]				
Coleoptera	*Xylotrechus arvicola*	[66]				
Diptera	*Drosophila suzukii*	[67,68]	[69]			
Acari	Spider mites	[70,71]				
